# Supplementary material for: The association between BMI and serum uric acid is partially mediated by gut microbiota
Source: Microbiol Spectr. 2023 Sep 25;11(5):e01140-23. doi: 10.1128/spectrum.01140-23 (PMC10581133; doi:10.1128/spectrum.01140-23)
Supplement: Supplemental material — Supplemental figures and tables. [file spectrum.01140-23-s0001.docx]

**Supplementary materials**

**Table S1. Characteristics of the study participants before and after imputing missing data**

| **Characteristics** | **Imputed data, N = 6,280** | **Raw data, N = 6,280** | **p-value** |
| --- | --- | --- | --- |
| BMI, Median (IQR) | 23.1 (20.9 – 25.5) | 23.1 (20.9 – 25.5) | >0.999 |
| UA(umol/L), Median (IQR) | 325 (270 – 391) | 325 (270 – 391) | >0.999 |
| Age, Median (IQR) | 53 (43 – 64) | 53 (43 – 64) | >0.999 |
| Gender(Female), n (%) | 3,488 (56) | 3,488 (56) | >0.999 |
| Education, Median (IQR) | 4.00 (2.00 – 4.00) | 4.00 (2.00 – 4.00) | >0.999 |
| Smoke now, n (%) | 1,625 (26) | 1,608 (26) | 0.942 |
| Sleep_time(min), Median (IQR) | 480 (420 – 480) | 480 (420 – 500) | 0.972 |
| SBP(mmHg), Median (IQR) | 128 (116 – 144) | 128 (116 – 144) | 0.994 |
| DBP(mmHg), Median (IQR) | 76 (70 – 84) | 77 (70 – 84) | 0.996 |
| HR(/min), Median (IQR) | 76 (69 – 84) | 76 (69 – 84) | 0.999 |
| FBG(mmol/L), Median (IQR) | 5.31 (4.92 – 5.80) | 5.31 (4.92 – 5.80) | 0.992 |
| TCHO(mmol/L), Median (IQR) | 5.22 (4.71 – 5.75) | 5.22 (4.71 – 5.75) | >0.999 |
| TG(mmol/L), Median (IQR) | 1.08 (0.76 – 1.60) | 1.08 (0.76 – 1.60) | >0.999 |
| HDL(mmol/L), Median (IQR) | 1.23 (1.01 – 1.48) | 1.23 (1.01 – 1.48) | 0.993 |
| LDL(mmol/L), Median (IQR) | 3.19 (2.62 – 3.82) | 3.19 (2.62 – 3.82) | >0.999 |
| Hb(g/L), Median (IQR) | 143 (131 – 155) | 143 (131 – 155) | >0.999 |
| ALT(U/L), Median (IQR) | 15 (11 – 22) | 15 (11 – 22) | 0.584 |
| BUN(mmol/L), Median (IQR) | 5.10 (4.28 – 6.05) | 5.09 (4.27 – 6.07) | 0.805 |
| Gout, n (%) | 206 (3.3) | 206 (3.3) | 0.924 |
| Bristol stool type, n (%) |  |  | >0.999 |
| 1 | 139 (2.2) | 139 (2.2) |  |
| 2 | 397 (6.3) | 397 (6.4) |  |
| 3 | 938 (15) | 938 (15) |  |
| 4 | 3,840 (61) | 3,759 (61) |  |
| 5 | 688 (11) | 688 (11) |  |
| 6 | 252 (4.0) | 252 (4.1) |  |
| 7 | 26 (0.4) | 26 (0.4) |  |
| Synbiotics use within 1 month, n (%) | 168 (2.7) | 168 (2.7) | 0.852 |
| Antibotics use within 1 month, n (%) | 375 (6.0) | 375 (6.1) | 0.741 |
| Drugs use within 3 days, n (%) | 1,062 (17) | 1,059 (17) | 0.601 |
| Grains(g), Median (IQR) | 109,500 (73,000 – 164,250) | 109,500 (72,308 – 164,250) | 0.677 |
| Vegetables(g), Median (IQR) | 109,500 (73,000 – 164,250) | 109,500 (73,000 – 164,250) | 0.617 |
| Fruits(g), Median (IQR) | 15,600 (4,800 – 36,500) | 15,600 (4,800 – 36,500) | 0.206 |
| Livestock meat(g), Median (IQR) | 36,500 (18,045 – 73,000) | 36,500 (17,095 – 73,000) | 0.341 |

Kruskal-Wallis rank sum test for continuous variables; Pearson's Chi-squared test for categorical variables.

**Table S2. The relationship between BMI and gut microbes**

| **Taxonomy** | *r* | *P* | FDR |
| --- | --- | --- | --- |
| Parabacteroides | -0.099540899 | 2.53E-15 | 4.26E-13 |
| Butyricimonas | -0.098296842 | 5.61E-15 | 4.71E-13 |
| Anaerotruncus | -0.094739352 | 5.15E-14 | 2.59E-12 |
| Bacteroides | -0.094389431 | 6.38E-14 | 2.59E-12 |
| Odoribacter | -0.094079386 | 7.71E-14 | 2.59E-12 |
| Christensenella | -0.090428078 | 6.80E-13 | 1.90E-11 |
| f__Christensenellaceae;g__ | -0.08757872 | 3.50E-12 | 8.41E-11 |
| f__Lachnospiraceae;g__Clostridium | -0.087216515 | 4.30E-12 | 9.03E-11 |
| Blautia | 0.084026999 | 2.52E-11 | 4.48E-10 |
| Dehalobacterium | -0.083920123 | 2.67E-11 | 4.48E-10 |
| Oscillospira | -0.083310724 | 3.71E-11 | 5.66E-10 |
| Ralstonia | 0.08229233 | 6.40E-11 | 8.96E-10 |
| Bradyrhizobium | 0.078122623 | 5.59E-10 | 7.22E-09 |
| Roseburia | 0.073802511 | 4.70E-09 | 5.64E-08 |
| f__Caulobacteraceae;g__ | 0.071715139 | 1.26E-08 | 1.41E-07 |
| Faecalibacterium | 0.070871076 | 1.87E-08 | 1.96E-07 |
| Megamonas | 0.06914428 | 4.10E-08 | 4.05E-07 |
| Dialister | 0.068611011 | 5.21E-08 | 4.86E-07 |
| f__Ruminococcaceae;g__Clostridium | -0.068380058 | 5.78E-08 | 5.11E-07 |
| Lachnospira | 0.06798694 | 6.88E-08 | 5.78E-07 |
| f__Ruminococcaceae;Other | -0.067857019 | 7.29E-08 | 5.83E-07 |
| Oxalobacter | -0.067545151 | 8.36E-08 | 6.38E-07 |
| p__Bacteroidetes;Other;Other;Other;Other | -0.067260534 | 9.47E-08 | 6.92E-07 |
| Ochrobactrum | 0.066992099 | 1.07E-07 | 7.46E-07 |
| f__Erysipelotrichaceae;g__Clostridium | -0.065907394 | 1.71E-07 | 1.15E-06 |
| o__Streptophyta;f__;g__ | 0.065551473 | 1.99E-07 | 1.28E-06 |
| f__Methylobacteriaceae;g__ | 0.06329301 | 5.14E-07 | 3.20E-06 |
| Sediminibacterium | 0.062166217 | 8.17E-07 | 4.90E-06 |
| f__Rikenellaceae;g__ | -0.061993248 | 8.77E-07 | 5.08E-06 |
| Coprobacillus | -0.061370489 | 1.13E-06 | 6.31E-06 |
| Alistipes | -0.060863546 | 1.38E-06 | 7.49E-06 |
| Anaerofilum | -0.059792949 | 2.11E-06 | 1.11E-05 |
| Sphingomonas | 0.05956593 | 2.31E-06 | 1.17E-05 |
| Butyricicoccus | 0.059250703 | 2.61E-06 | 1.29E-05 |
| Turicibacter | -0.058929977 | 2.96E-06 | 1.42E-05 |
| f__Erysipelotrichaceae;Other | -0.057356502 | 5.39E-06 | 2.52E-05 |
| f__Chitinophagaceae;g__ | 0.05722008 | 5.68E-06 | 2.58E-05 |
| f__Comamonadaceae;Other | 0.055487361 | 1.08E-05 | 4.78E-05 |
| Coprococcus | 0.055341156 | 1.14E-05 | 4.91E-05 |
| Devosia | 0.055173596 | 1.21E-05 | 5.09E-05 |
| Pseudidiomarina | 0.053682926 | 2.07E-05 | 8.49E-05 |
| f__[Barnesiellaceae];g__ | -0.053147766 | 2.50E-05 | 0.000100177 |
| Dorea | 0.053059695 | 2.58E-05 | 0.000100925 |
| Methylobacterium | 0.052598641 | 3.04E-05 | 0.000115906 |
| Defluviitalea | -0.052468262 | 3.18E-05 | 0.000118594 |
| Epulopiscium | -0.052358227 | 3.30E-05 | 0.000120538 |
| Holdemania | -0.051833094 | 3.96E-05 | 0.000141449 |
| Akkermansia | -0.051276545 | 4.79E-05 | 0.00016757 |
| o__Clostridiales;Other;Other | -0.050570086 | 6.08E-05 | 0.000208493 |
| Synergistes | -0.049286878 | 9.32E-05 | 0.000313039 |
| Rheinheimera | 0.049041155 | 0.000100983 | 0.000332649 |
| Halomonas | 0.048293564 | 0.000128742 | 0.000415935 |
| Delftia | 0.046922181 | 0.000199251 | 0.00063159 |
| f__Lachnospiraceae;g__ | 0.04479751 | 0.000383348 | 0.001192638 |
| Desulfovibrio | -0.044389854 | 0.00043329 | 0.001323505 |
| Ruminococcus | 0.04421477 | 0.000456551 | 0.001369653 |
| Lupinus | 0.044146374 | 0.00046595 | 0.001373327 |
| Methanobrevibacter | -0.043946565 | 0.000494452 | 0.001432207 |
| Pseudoramibacter_Eubacterium | -0.04296123 | 0.000660341 | 0.001880293 |
| f__Erysipelotrichaceae;g__ | 0.04273767 | 0.000704573 | 0.001959567 |
| f__[Paraprevotellaceae];g__ | -0.042671756 | 0.00071813 | 0.001959567 |
| Acinetobacter | 0.04264753 | 0.000723174 | 0.001959567 |
| Arthrobacter | 0.041184181 | 0.001096547 | 0.002924125 |
| WAL_1855D | -0.041019152 | 0.001148336 | 0.003014383 |
| Pseudomonas | 0.040168768 | 0.001452894 | 0.003755171 |
| Sutterella | 0.040068288 | 0.001493422 | 0.003801439 |
| f__Coriobacteriaceae;Other | -0.039733151 | 0.001636249 | 0.004102834 |
| Mitsuokella | 0.038258542 | 0.002426432 | 0.005994713 |
| f__Clostridiaceae;g__02d06 | -0.037989081 | 0.002603974 | 0.006340112 |
| Porphyromonas | -0.037778593 | 0.002750836 | 0.006602005 |
| f__Fusobacteriaceae;Other | 0.036692867 | 0.003635636 | 0.008602631 |
| Pyramidobacter | -0.036219669 | 0.004096654 | 0.00955886 |
| Parvimonas | -0.035880776 | 0.004458752 | 0.010261237 |
| Blastomonas | 0.035746707 | 0.004609831 | 0.010465563 |
| Methylotenera | 0.034675154 | 0.005994001 | 0.013426562 |
| Brevibacterium | 0.033999487 | 0.00704903 | 0.015582066 |
| f__Desulfovibrionaceae;g__ | -0.033484348 | 0.007962341 | 0.017356475 |
| Cloacibacillus | -0.033433308 | 0.008058364 | 0.017356475 |
| Collinsella | 0.032998092 | 0.008920181 | 0.0189695 |
| [Ruminococcus] | 0.032674177 | 0.009614067 | 0.02018954 |
| o__MLE1-12;f__;g__ | -0.032528984 | 0.009940421 | 0.020437414 |
| [Prevotella] | 0.032513673 | 0.009975404 | 0.020437414 |
| f__Clostridiaceae;Other | -0.03238025 | 0.010284927 | 0.020817683 |
| Prevotella | 0.03186394 | 0.011564737 | 0.023129474 |
| Prauserella | 0.031772266 | 0.011806193 | 0.023334593 |
| Herbaspirillum | 0.030457072 | 0.015796113 | 0.030787718 |
| f__[Mogibacteriaceae];g__ | -0.030388328 | 0.016033965 | 0.030787718 |
| Bifidobacterium | -0.03036171 | 0.0161269 | 0.030787718 |
| Comamonas | 0.028511335 | 0.02386381 | 0.045046293 |
| f__Prevotellaceae;g__ | -0.02825848 | 0.025138541 | 0.046925277 |
| Chryseobacterium | 0.02790189 | 0.02703616 | 0.04991291 |
| Peptococcus | -0.02753767 | 0.029100738 | 0.053140478 |
| Stenotrophomonas | 0.027417639 | 0.029810207 | 0.053850696 |
| Peptoniphilus | -0.027354342 | 0.030190294 | 0.053957121 |
| Flavobacterium | 0.027153209 | 0.031425801 | 0.055574048 |
| Psychrobacter | 0.027002821 | 0.032377704 | 0.056660981 |
| f__Clostridiaceae;g__ | -0.026649843 | 0.03470937 | 0.060115197 |
| f__Phyllobacteriaceae;g__ | 0.026509316 | 0.035676775 | 0.061160185 |
| Fusobacterium | -0.026210241 | 0.037812389 | 0.064166478 |
| Limnohabitans | 0.025474388 | 0.043534161 | 0.073137391 |
| Novosphingobium | 0.025058478 | 0.047080131 | 0.078311504 |
| f__Gemellaceae;g__ | -0.02384833 | 0.058804538 | 0.096854533 |

Model was adjusted for age, gender, TG, BUN, HDL, Hb, LDL, FBG, SBP, DBP, ALT, TCHO, Grains, Livestock_meat and Fruits.

**Table S3. The association between gut microbes and SUA**

| **Taxonomy** | *r* | 2.50% | 97.50% | *P* | FDR |
| --- | --- | --- | --- | --- | --- |
| Odoribacter | -1433.808778 | -2854.827102 | -12.79045389 | 0.047973864 | 0.1886972 |
| Megamonas | 58.38129001 | 4.272747215 | 112.4898328 | 0.034457159 | 0.174410195 |
| f__Erysipelotrichaceae;g__Clostridium | 401.0049279 | 9.198506419 | 792.8113495 | 0.044860138 | 0.182534353 |
| f__Rikenellaceae;g_ | -86.16301104 | -169.7150556 | -2.61096652 | 0.043259888 | 0.18230953 |
| f__Chitinophagaceae;g_ | -3010.987595 | -5773.862399 | -248.1127911 | 0.032686669 | 0.174410195 |
| f__Comamonadaceae;Other | 3334.621627 | 1576.915894 | 5092.327361 | 0.000201738 | 0.002380509 |
| Synergistes | 883.7802197 | 53.53521826 | 1714.025221 | 0.036951312 | 0.174410195 |
| Acinetobacter | 185.6300133 | 21.74857256 | 349.5114541 | 0.026420716 | 0.171331426 |
| f__Coriobacteriaceae;Other | -9603.89732 | -18188.05477 | -1019.739867 | 0.028328391 | 0.171331426 |
| Pyramidobacter | -1200.971493 | -2145.989677 | -255.9533085 | 0.012753785 | 0.100329772 |
| [Ruminococcus] | 92.47164875 | 16.97357832 | 167.9697192 | 0.01637633 | 0.120775434 |
| [Prevotella] | 127.0400469 | 5.390934547 | 248.6891593 | 0.040678535 | 0.177780262 |
| f__Prevotellaceae;g_ | 2426.904314 | 247.9909998 | 4605.817628 | 0.029039225 | 0.171331426 |
| Chryseobacterium | 4866.509097 | 229.6649955 | 9503.353199 | 0.039685326 | 0.177780262 |
| f__Phyllobacteriaceae;g_ | -7387.977774 | -14287.42478 | -488.5307712 | 0.035843737 | 0.174410195 |
| Novosphingobium | 14299.27266 | 1182.772939 | 27415.77237 | 0.032627294 | 0.174410195 |

Model was adjusted for age, gender, TG, BUN, HDL, Hb, LDL, FBG, SBP, DBP, ALT, TCHO, Grains, Livestock_meat and Fruits.

**Table S4. The mediation analysis of gut microbes(phylum) on the association between BMI and SUA**

| Taxon | Level | Indirect effect | 95%CI | P |
| --- | --- | --- | --- | --- |
| Bacteroidetes | phylum | 0.028 | （-0.011, 0.072） | 0.176 |
| Firmicutes | phylum | -0.027 | （-0.071, 0.014） | 0.203 |
| Proteobacteria | phylum | 0.046 | （0.013, 0.093） | 0.021 |
| Verrucomicrobia | phylum | 0.022 | （-0.014, 0.069） | 0.284 |

Model was adjusted for age, gender, TG, BUN, HDL, Hb, LDL, FBG, SBP, DBP, ALT, TCHO, Grains, Livestock_meat and Fruits.

**Table S5. The mediation effects (%, Proportion mediated) of bacterial taxa on the associations between BMI and SUA**

| Taxon | Level | Indirect effect | Mediation  (%) | 95%CI | P |
| --- | --- | --- | --- | --- | --- |
| g__Odoribacter | Genus | 0.061 | 1.25 | (0.026, 0.112) | 0.005 |
| f__Christensenellaceae;g_ | Genus | 0.027 | 0.55 | (0.005, 0.053) | 0.037 |
| g__Dehalobacterium | Genus | 0.042 | 0.86 | (0.014, 0.073) | 0.01 |
| g__Oscillospira | Genus | 0.06 | 1.23 | (0.012, 0.112) | 0.015 |
| g__Ralstonia | Genus | 0.135 | 2.76 | (0.069, 0.203) | <0.001 |
| g__Bradyrhizobium | Genus | 0.083 | 1.7 | (0.028, 0.149) | 0.01 |
| f__Caulobacteraceae;g_ | Genus | 0.098 | 2 | (0.040, 0.165) | 0.002 |
| g__Faecalibacterium | Genus | -0.057 | 1.17 | (-0.116, -0.010) | 0.028 |
| g__Ochrobactrum | Genus | 0.069 | 1.41 | (0.024, 0.129) | 0.009 |
| g__Clostridium | Genus | -0.042 | 0.86 | (-0.071, -0.017) | 0.002 |
| f__Methylobacteriaceae | Genus | 0.053 | 1.09 | (0.019, 0.090) | 0.005 |
| g__Sediminibacterium | Genus | 0.05 | 1.02 | (0.006, 0.100) | 0.039 |
| f__Rikenellaceae;g_ | Genus | 0.053 | 1.09 | (0.014, 0.098) | 0.014 |
| f__Comamonadaceae;Other | Genus | 0.133 | 2.72 | (0.072, 0.203) | <0.001 |
| g__Coprococcus | Genus | -0.047 | 0.96 | (-0.094, -0.011) | 0.03 |
| g__Devosia | Genus | 0.044 | 0.9 | (0.011, 0.090) | 0.037 |
| g__Pseudidiomarina | Genus | 0.061 | 1.25 | (0.019, 0.113) | 0.01 |
| f__[Barnesiellaceae];g_ | Genus | 0.05 | 1.02 | (0.020, 0.088) | 0.005 |
| g__Rheinheimera | Genus | 0.041 | 0.84 | (0.007, 0.087) | 0.049 |
| g__Halomonas | Genus | 0.072 | 1.47 | (0.025, 0.123) | 0.005 |
| g__Delftia | Genus | 0.06 | 1.23 | (0.021, 0.111) | 0.012 |
| g__Methanobrevibacter | Genus | 0.031 | 0.63 | (0.007, 0.064) | 0.031 |
| g__Methylotenera | Genus | 0.04 | 0.82 | (0.008, 0.083) | 0.047 |
| g__Bifidobacterium | Genus | -0.037 | 0.76 | (-0.071, -0.007) | 0.026 |
| g__Novosphingobium | Genus | 0.047 | 0.96 | (0.010, 0.097) | 0.033 |

The mediation analyses adjusted for age, gender, TG, BUN, HDL, Hb, LDL, FBG, SBP, DBP, ALT, TCHO, Grains, Livestock_meat and Fruits.

**Table S6. Significant mediated effect genus reproduced in the partially random sampling analyses**

| Taxon | 6280 | 5280 | 4280 | 3280 |
| --- | --- | --- | --- | --- |
| g__Odoribacter | 1 | 1 | 1 | 1 |
| f__Christensenellaceae;g_ | 1 | 1 | 1 | 1 |
| g__Dehalobacterium | 1 | 1 | 1 | 1 |
| g__Oscillospira | 1 | 1 | 1 | 1 |
| g__Ralstonia | 1 | 1 | 1 | 1 |
| g__Bradyrhizobium | 1 | 1 | 1 | 1 |
| f__Caulobacteraceae;g_ | 1 | 1 | 1 | 1 |
| g__Faecalibacterium | 1 | 1 | 1 | 0 |
| g__Ochrobactrum | 1 | 1 | 1 | 1 |
| g__Clostridium | 1 | 1 | 1 | 0 |
| f__Methylobacteriaceae | 1 | 0 | 0 | 0 |
| g__Sediminibacterium | 1 | 1 | 1 | 0 |
| f__Rikenellaceae;g_ | 1 | 1 | 1 | 1 |
| f__Comamonadaceae;Other | 1 | 0 | 0 | 0 |
| g__Coprococcus | 1 | 1 | 1 | 1 |
| g__Devosia | 1 | 0 | 0 | 0 |
| g__Pseudidiomarina | 1 | 0 | 0 | 0 |
| f__[Barnesiellaceae];g_ | 1 | 0 | 0 | 0 |
| g__Rheinheimera | 1 | 1 | 0 | 0 |
| g__Halomonas | 1 | 1 | 0 | 0 |
| g__Delftia | 1 | 0 | 0 | 0 |
| g__Methanobrevibacter | 1 | 0 | 0 | 0 |
| g__Methylotenera | 1 | 0 | 0 | 0 |
| g__Bifidobacterium | 1 | 0 | 1 | 0 |
| g__Novosphingobium | 1 | 0 | 0 | 0 |

Model was adjusted for age, gender, TG, BUN, HDL, Hb, LDL, FBG, SBP, DBP, ALT, TCHO, Grains, Livestock_meat and Fruits. “1” means “Yes” while “0” means “No”.

**Supplementary figures**

**FigS1**

**
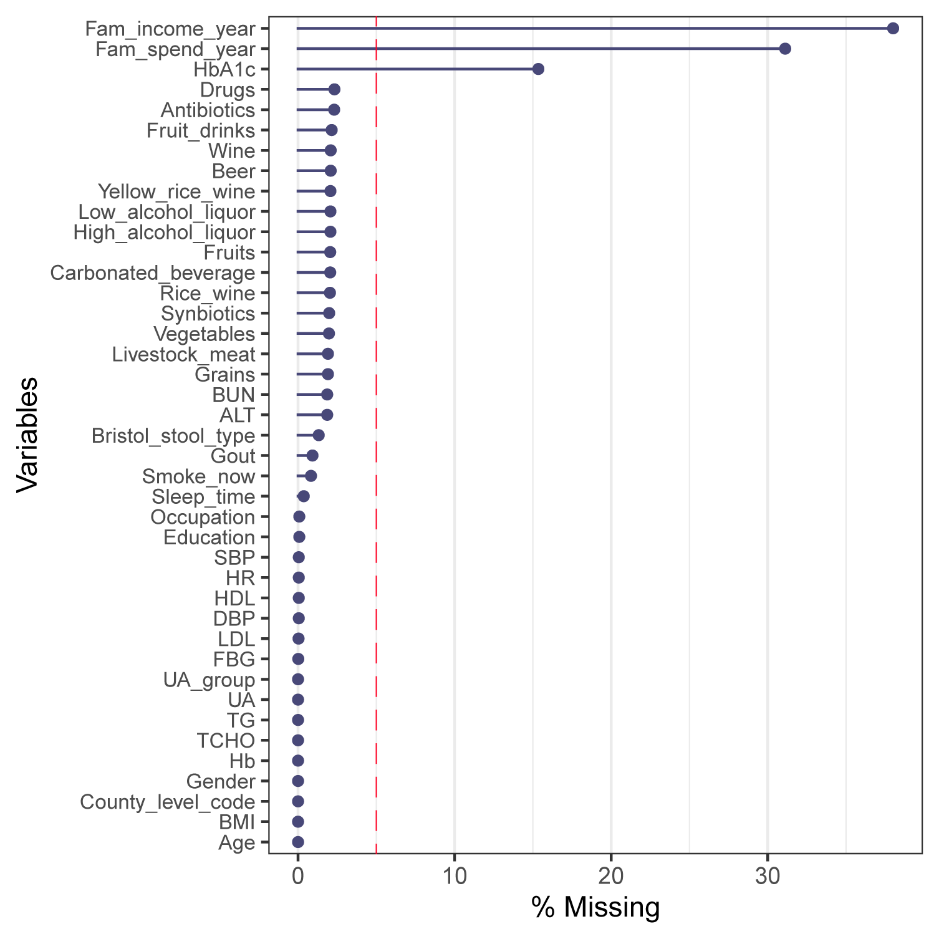
**

**FigS1. The missing rate for the host baseline characteristics.** The horizontal axis represents the missing rate for each baseline characteristic. Characteristics with a missing rate lower than 5% (those below the red dashed line) are remained in the downstream analysis.

**FigS2**

**
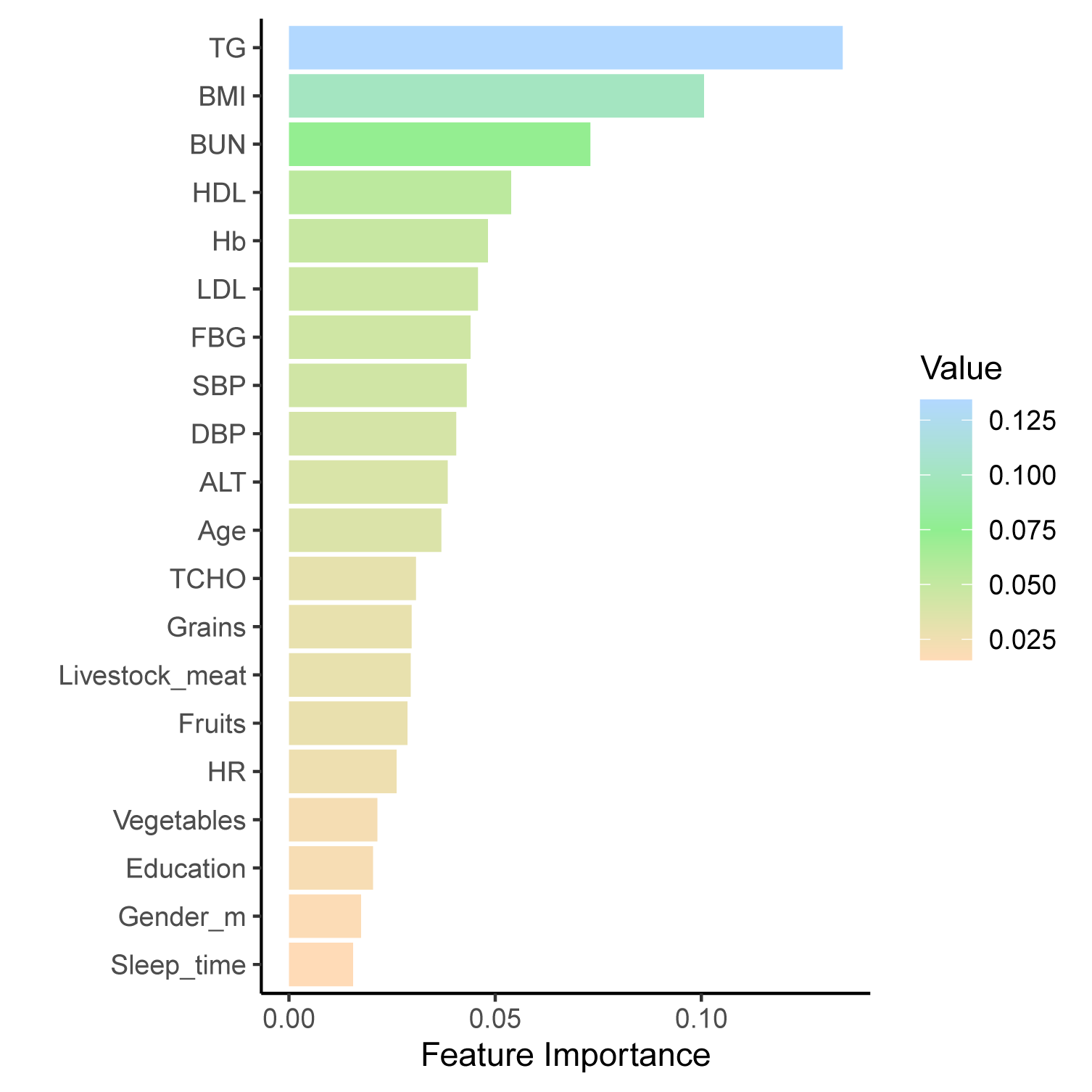
**

**FigS2. The feature importance of top 20 baseline characteristics associated with risks of HUA.** The global feature importance calculated by the random forest’s built-in Gini impurity-based method is depicted in the bar plot.

**FigS3**

**
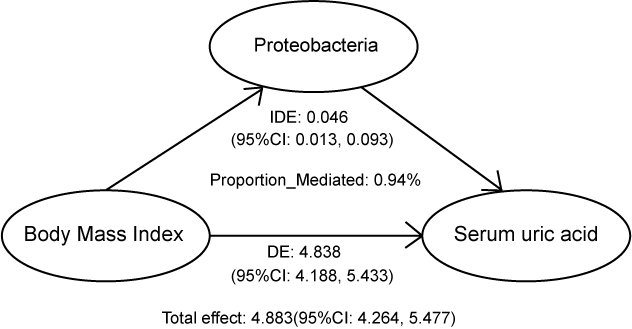
**

**FigS3. The mediation effect of *Proteobacteria* on the association between BMI and SUA.** Model was adjusted for age, gender, TG, BUN, HDL, Hb, LDL, FBG, SBP, DBP, ALT, TCHO, Grains, Livestock_meat and Fruits.

**FigS4**


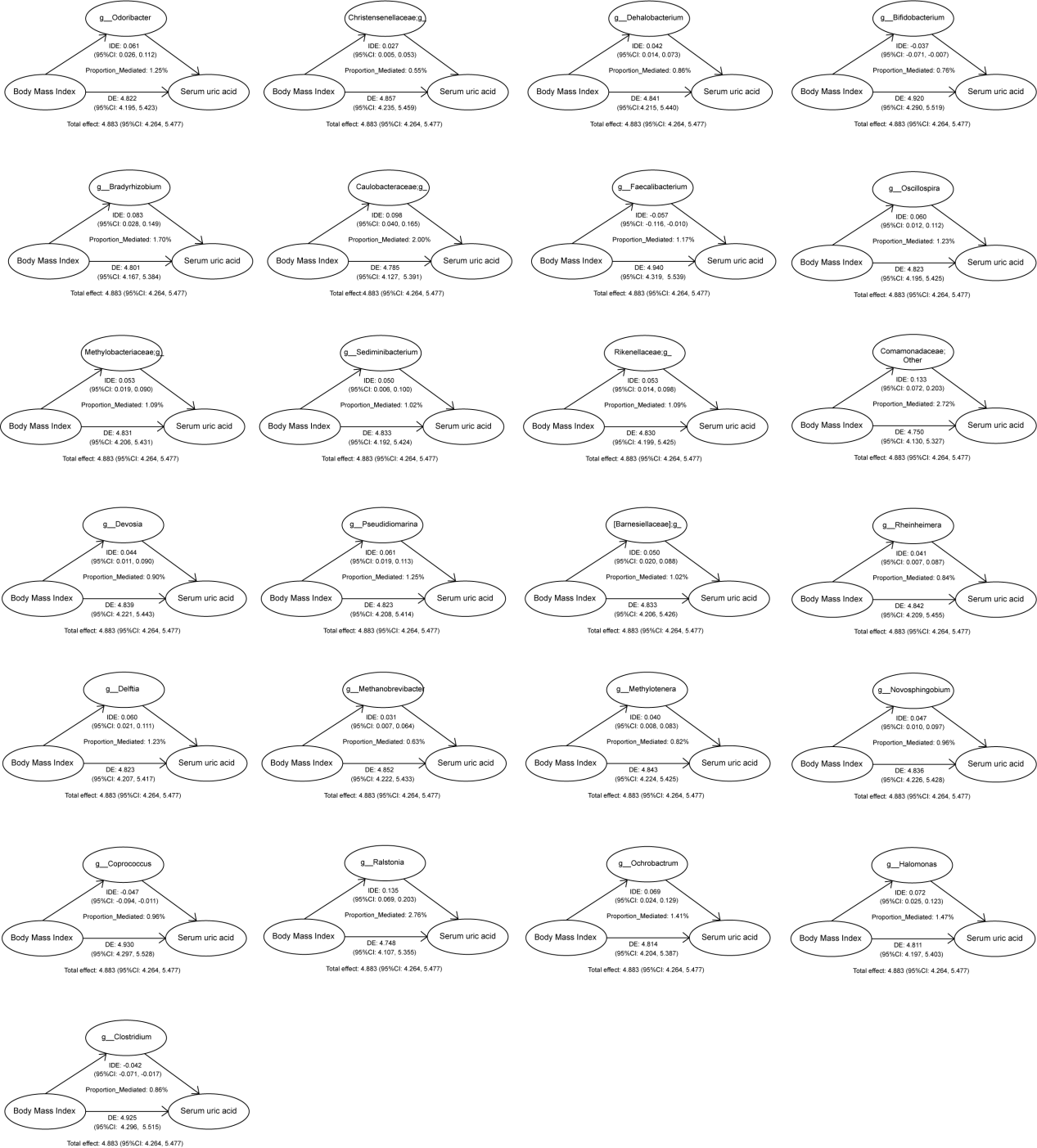


**FigS4. The mediation effects of the gut microbes on the association between BMI and SUA.** Model was adjusted for age, gender, TG, BUN, HDL, Hb, LDL, FBG, SBP, DBP, ALT, TCHO, Grains, Livestock_meat and Fruits.

**FigS5**

**
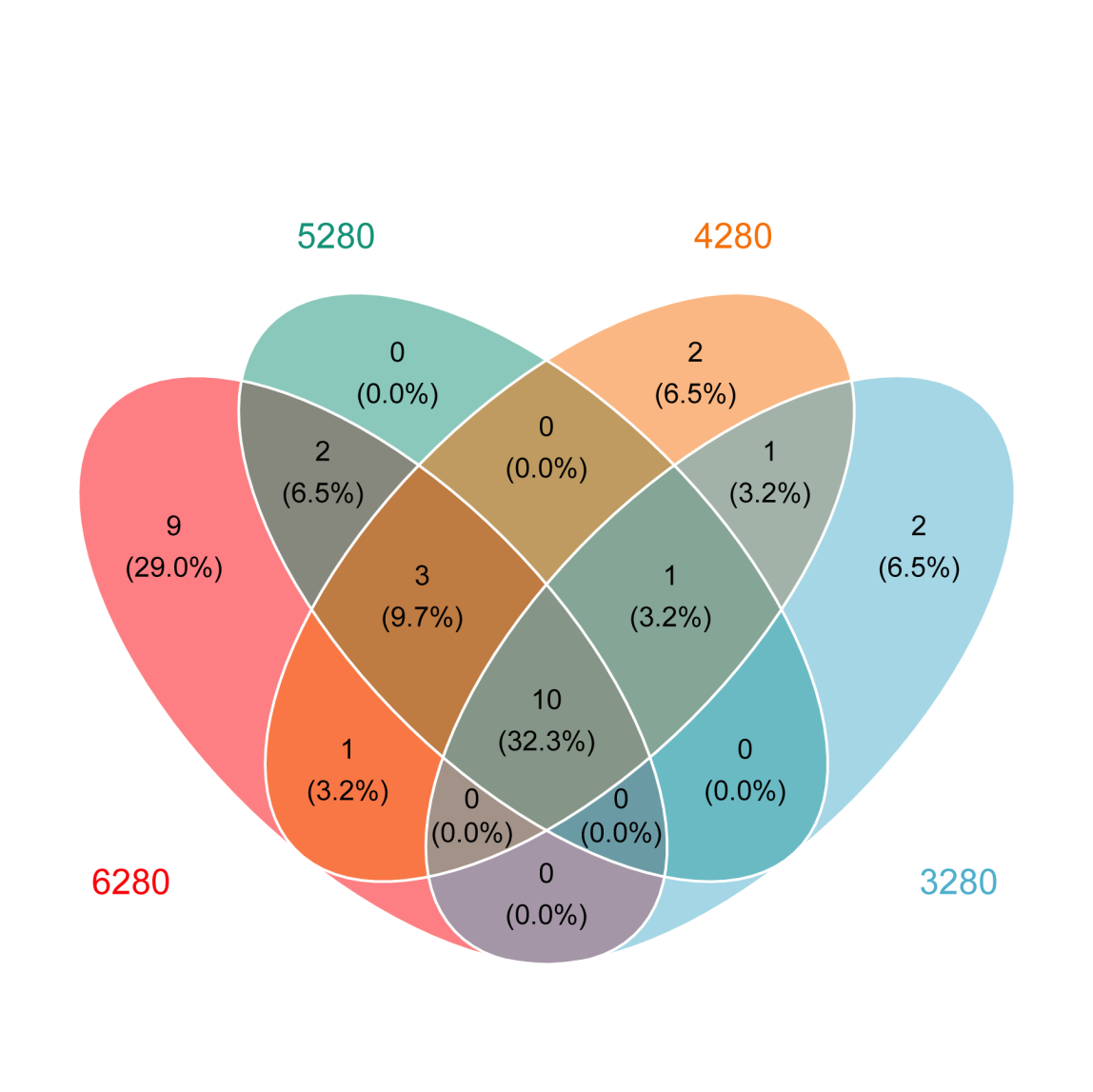
**

**FigS5. Significant mediated effect genus in random partial sampling groups.** The Venn plot illustrates the number and percent of the overlapping genus in different sample size groups. Model was adjusted for age, gender, TG, BUN, HDL, Hb, LDL, FBG, SBP, DBP, ALT, TCHO, Grains, Livestock_meat and Fruits.
